# Fabrication of graphene oxide/montmorillonite nanocomposite flexible thin films with improved gas-barrier properties †

**DOI:** 10.1039/c8ra08232d

**Published:** 2018-11-20

**Authors:** Se Jung Kim, Tan young Kim, Byung Hyun Kang, Gun-Hwan Lee, Byeong-Kwon Ju

**Affiliations:** Display and Nanosystem Laboratory, Department of Electrical Engineering 145, Anam-ro, Seongbuk-gu Seoul 02841 Republic of Korea bkju@korea.ac.kr; Korea Institute of Materials Science, Surface Technology Division 797 Changwondaero Sungsangu Changwon Gyeongnam Korea ghlee@kims.re.kr

## Abstract

Nanocomposites are potential substitutes for inorganic materials in fabricating flexible gas-barrier thin films. In this study, two nanocomposites are used to form a flexible gas-barrier film that shows improved flexibility and a decreased water vapor transmission rate (WVTR), thereby extending the diffusion path length for gas molecules. The nanoclay materials used for the flexible gas-barrier thin film are Na^+^-montmorillonite (MMT) and graphene oxide (GO). A flexible gas-barrier thin film was fabricated using a layer-by-layer (LBL) deposition method, exploiting electronic bonding under non-vacuum conditions. The WVTR of the film, in which each layer was laminated by LBL assembly, was analyzed by Ca-test and the oxygen transmission rate (OTR) was analyzed by MOCON. When GO and MMT are used together, they fill each other's vacancies and form a gas-barrier film with high optical transmittance and the improved WVTR of 3.1 × 10^−3^ g per m^2^ per day without a large increase in thickness compared to barrier films produced with GO or MMT alone. Thus, this film has potential applicability as a barrier film in flexible electronic devices.

## Abstract 2

Nanocomposites are potential substitutes for inorganic materials in fabricating flexible gas-barrier thin films.

## Graphical abstract

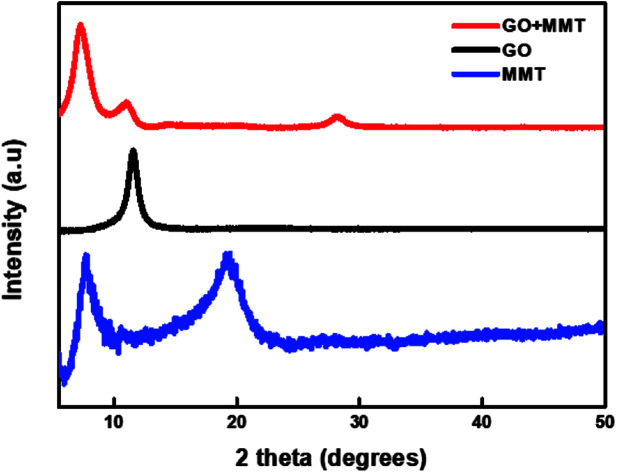

## Introduction

When flexibility is required in new electronic devices, flexible gas-barrier films that prevent water vapor transmission within such devices are of significant research interest.^1^ Previous developed technologies have used both organic and inorganic films with SiO_*x*_ or Al_*x*_O_*y*_ multilayered structures.^2^ The inorganic layers were typically fabricated by vacuum processes, such as chemical vapor deposition, atomic layer deposition, and sputtering.^3–5^ However, vacuum processing has the disadvantages of low production efficiency and high production costs; inorganic layers also frequently crack under bending stresses, which permits H_2_O and O_2_ to flow through flexible electronic devices. H_2_O and O_2_ react electrochemically with the cathode as active metals, forming additional H_2_ gas inside the device. This H_2_ gas forms bubbles at the cathode, which destroys the device.^6^

For this reason, a mixture of GO and other materials such as polymer or nano clay which can be laminated by a non-vacuum process has seen increased interest as a good alternative to standard vacuum-processed inorganic layers.^7–9^ In this study, a flexible gas-barrier thin film is fabricated using the layer-by-layer (LBL) deposition method, based on graphene oxide, nano clay materials of Na^+^-montmorillonite (MMT) and the two polymers of polydiallydimethylammonium chloride (PDDA) and poly(vinyl alcohol) (PVA).^10–12^ Nanoclay layers can be fabricated through LBL assembly processing.

LBL refers to the general process of laminating using hydrogen bonding, covalent bonding, or electrostatic attraction under non-vacuum conditions. Therefore, gas-barrier films fabricated through LBL processes possess steady structures formed in a cost-effective and short tact time.^13–15^ We fabricated a gas-barrier thin film with a decreased WVTR and OTR compared to a film having the same number of layers by combining the nanoclays with the polymers to form adhesion layers. GO has a large aspect ratio and MMT is a plate-shaped material and both materials have good dispersibility in water, so they are suitable materials for lengthening the moisture permeation pathway in a gas-barrier thin film.^16–19^ The reaction between MMT and PVA has a negative charge,^20^ while that of GO and PDDA has a positive charge. The fabricated flexible gas-barrier films showed good transparency and improved WVTR characteristics because the two materials were alternately laminated using electrostatic attraction in the LBL process. The water vapor transmission properties of the flexible gas-barrier thin films were analyzed by Ca-test. A bending test confirmed that this flexible gas-barrier thin film could be applied to flexible devices.

## Experimental

### Gas-barrier film fabrication

GO (500 mg L^−1^) was purchased from the Graphene Supermarket. A mixture of 0.01 wt% GO in 200 mL of deionized (DI) water was magnetically stirred (450–550 rpm) for 24 h to disperse the GO uniformly. A solution of 0.02 wt% PDDA (*M*_w_ = 200 000–350 000, 20 wt% H_2_O) in DI water was prepared by magnetic stirring for 24 h. A mixture of the GO and PDDA solutions was magnetically stirred for 24 h to combine by the electrostatic attractions between the functional groups of the GO surface and the PDDA. In solution, yielding sheets of GO with positively charged surfaces.^21,22^ The PDDA(GO) solution was adjusted to the pH of 10 using 1 M NaOH.^23^ MMT was dispersed as a 0.05 wt% suspension in DI water by magnetic stirring for 24 h and then centrifuged at 4500 rpm for 1 h. After the first centrifugation, large MMT particles were dispersed at the bottom of the solution. These large submerged MMT particles were extracted and centrifuged at 1700 rpm for 15 min to yield MMT particles of uniform size between 2 and 4 μm. The resulting solution was magnetically stirred for 24 h and mixed in a 3 : 1 volumetric ratio with 0.5 wt% PVA (*M*_w_ = 30 000–70 000, 87–90% hydrolyzed, purchased from Sigma-Aldrich). The mixture was magnetically stirred for 24 h at 85 °C and stirred for another 24 h at room temperature in order to absorb the PVA between the MMT layers in solution. The PVA(MMT) solution was adjusted to a pH of 3.5 using 1 M HCl and comprised negatively charged complexes.

**Scheme 1:**
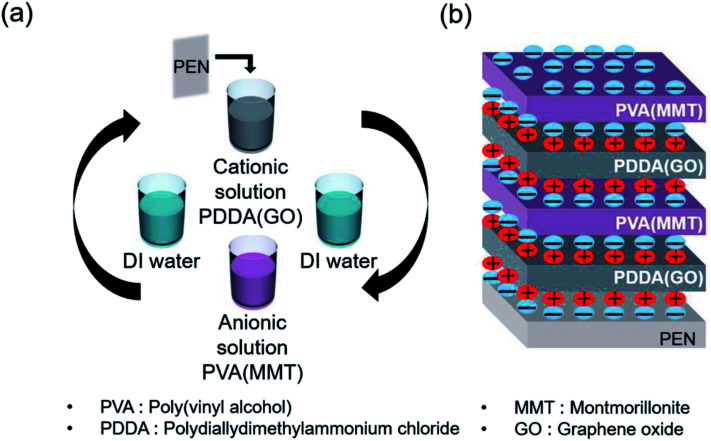
(a) Sequence of fabrication of the PDDA (GO)/PVA (MMT) film by LBL process. (b) Structure of the fabricated PDDA (GO)/PVA (MMT) film by electrical bonding.

Scheme 1(a) shows the gas-barrier thin films fabricated by the LBL process. A cleaned polyethylene naphthalate (PEN) film with a thickness of 125 μm (purchased from DuPont Teijin) was treated by ultraviolet (UV) ozone for 20 min in order to form OH- radicals on its surface. The PEN substrate was dipped in the PDDA(GO) solution for 10 min at room temperature by an automated dipping system. Next, the substrate was rinsed with DI water for 3 min and dried with filtered air. Afterward, the substrate was dipped in the PVA(MMT) solution for 10 min. After the final rinsing and drying, a single PDDA(GO)/PVA(MMT) bilayer was formed on the PEN substrate. For the second bilayer, the process was repeated with the dipping times in the PDDA(GO) and PVA(MMT) solutions reduced to 1 min. The film thickness, light transmittance, and WVTR of the gas-barrier thin films could be varied by changing the number of bilayers, as defined by the number of times the LBL dipping process was repeated.

### WVTR and OTR

The performances of the gas-barrier films fabricated by LBL processing on a PEN substrate were evaluated by measuring the WVTR using the Ca-test and the OTR using the MOCON OX-TRAN 2/21 MH (as specified in ASTM D-3985) at 23 °C and 50% RH. The Ca-test is a conventional method for measuring WVTR, with a minimum rate of 10^−6^ g per m^2^ per day. Ca is highly sensitive to water and water vapor; when it reacts with water vapor passing through a gas-barrier film, calcium oxide is formed. This oxidizes the Ca; therefore, an insulating film experiences an increase in resistance value under an applied constant voltage, which can be observed by monitoring the decrease in current through the film.

### Analysis using zeta-potential, TEM, SEM, XRD, AFM and UV-vis spectrophotometry

To characterize the electrostatic attraction between the PDDA(GO) and PVA(MMT) solutions, a zeta-potential analyzer (ELSZ-1000, Otsuka Electronics) was used. The focused ion-beam (FIB) technique was used to prepare cross-sections of the gas-barrier films for analysis by transmission electron microscopy (TEM) and scanning electron microscopy (SEM). The interlayer spacings of GO, MMT, and GO–MMT were examined by X-ray diffraction (XRD) analysis with a Rigaku SmartLab diffractometer operated at 45 kV voltage and 200 mA current using Cu Kα (*λ* = 0.154 nm) radiation. The surface roughness values of the films were analyzed by atomic force microscopy (AFM, XE 100, Park systems). The light transmittance values of the films were measured using an ultraviolet-visible (UV-vis) spectrophotometer (Cary 5000, Varian Instruments) in the wavelength range from 400 to 800 nm.

## Results and discussion

The PEN substrate, surface-treated by UV ozone, is alternately dipped into the PDDA(GO) and PVA(MMT) solutions. The surface charge of each solution was analyzed by the zeta-potential analyzer. We adjusted the pH of each solution and compared the zeta-potential values to obtain the appropriate adhesion. The zeta-potential value of the PDDA(GO) solution increases as the pH is decreased. At low pH, the carboxyl groups on the surface of GO are protonated and increase the number of COO^−^ groups; thus, the repulsive forces between the GO sheets are reduced.^24^ The experiment was performed under acidic conditions at a pH of 3.5, appropriate for the fabrication of gas-barrier films, because very low pH values induce insufficient charge densities and consequently disturb the assembly of each film layer.^24^ For the PVA(MMT) solution, the zeta-potential decreases as the pH increases. Thus, the pH of the PVA(MMT) solution is adjusted to 10.^25^ The PDDA(GO) solution has a positive surface charge of 39.73 mV, while PVA(MMT) has the negative surface charge of −10.98 mV.

**Fig. 1:**
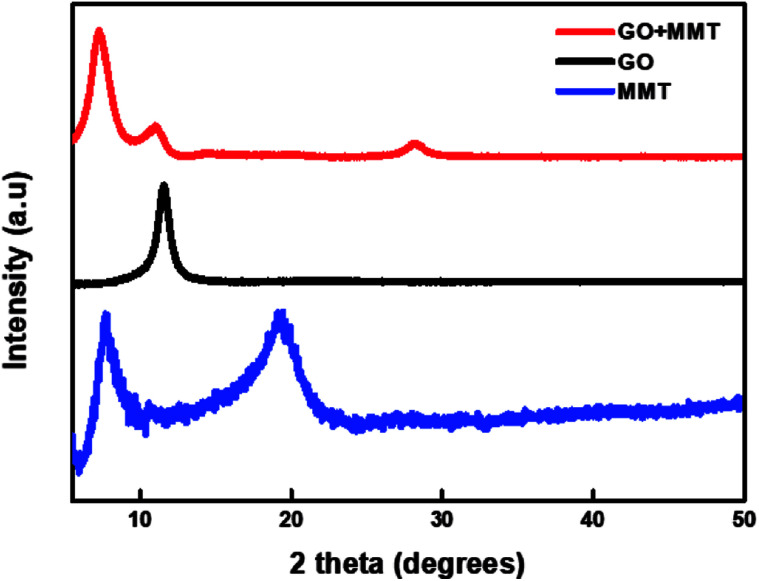
XRD patterns of GO, MMT, and GO + MMT.

Through XRD analysis confirmed that the incorporation between the GO and MMT. Fig. 1 shows the XRD patterns of GO, MMT, and GO–MMT. For GO, an obvious diffraction peak appears at 2*θ* = 11.56°, which corresponds to the *d*-spacing of 0.76 nm. MMT yields two intense diffraction peaks at 2*θ* = 7.7° and 19.1°, indicating interlayer distances of 1.15 and 0.46 nm, respectively. In the XRD profile of the GO–MMT layer, diffraction peaks are located at 2*θ* = 7.21° and 26.92°. The shift of the MMT peak from 7.7° to 7.21° indicates that MMT and GO are incorporated.^26,27^

**Fig. 2:**
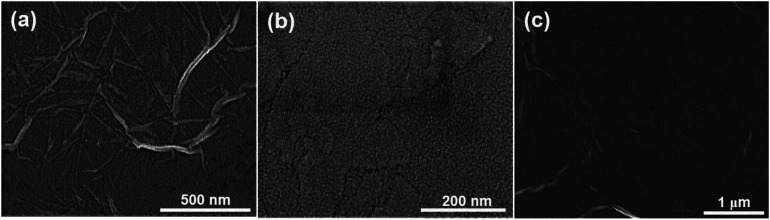
SEM images of (a) PDDA(GO), (b) PVA(MMT), and (c) PDDA(GO)/PVA(MMT) film.

Fig. 2 shows SEM micrographs of the PDDA(GO)/PVA(MMT) multilayer coated films, confirming that each layer is successfully assembled by electrostatic bonding. Fig. 2(a) and (b) show that each PDDA(GO) and PVA(MMT) layer is well stacked through the LBL process. Fig. 2(c) confirms that the PDDA(GO) and PVA(MMT) are laminated alternately with less roughness compared to a film of only laminated GO or MMT.

**Fig. 3:**
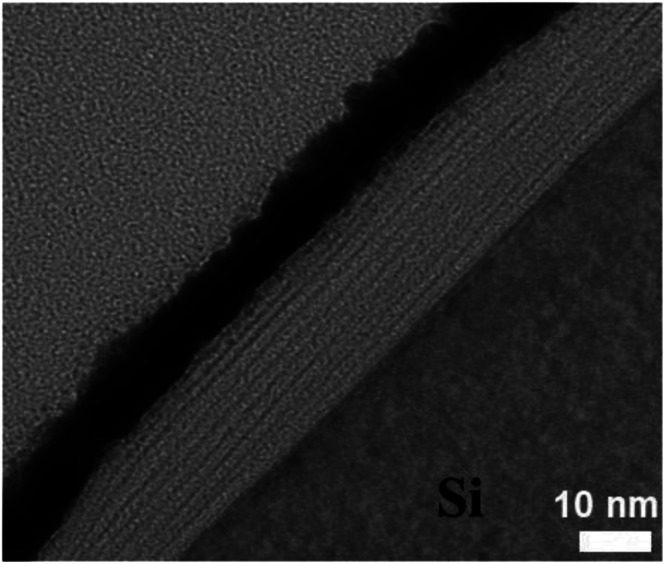
Cross-sectional TEM image of PDDA(GO)/PVA(MMT) film.

Fig. 3 shows TEM cross section image of the PDDA(GO)/PVA(MMT) composite films. The layer thicknesses in the gas barrier thin films are in the single-nanometer range. In the TEM image, the white part is PDDA(GO) and the black part is PVA(MMT).

**Scheme 2:**
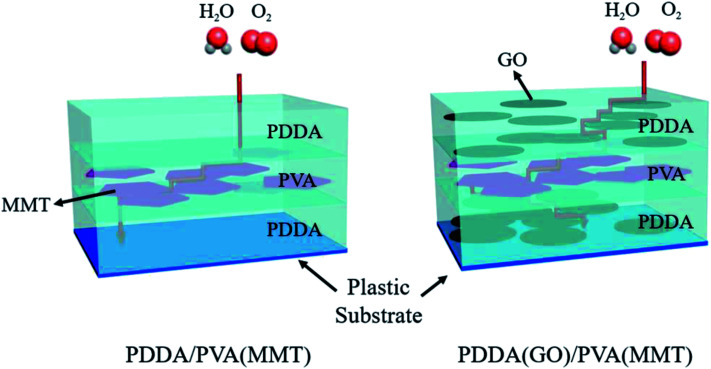
Schematics of PDDA/PVA(MMT) and PDDA(GO)/PVA(MMT) diffusion path.

Scheme 2 shows that GO mixed PDDA nanocomposites increased the diffusion path compared to untreated nanocomposites. It also confirmed that the diffusion path is extends without the increase of thickness when GO was mixed with the PDDA through the SEM image comparing the thickness of the gas-barrier films. Furthermore, when GO and MMT are alternately laminated, they materials are more stably bonded by hydrogen-bonding or crosslinking effects between GO and MMT, thus filling the vacancies formed when the layers are laminated individually and blocking pathways for H_2_O permeation.^28–32^

**Fig. 4:**
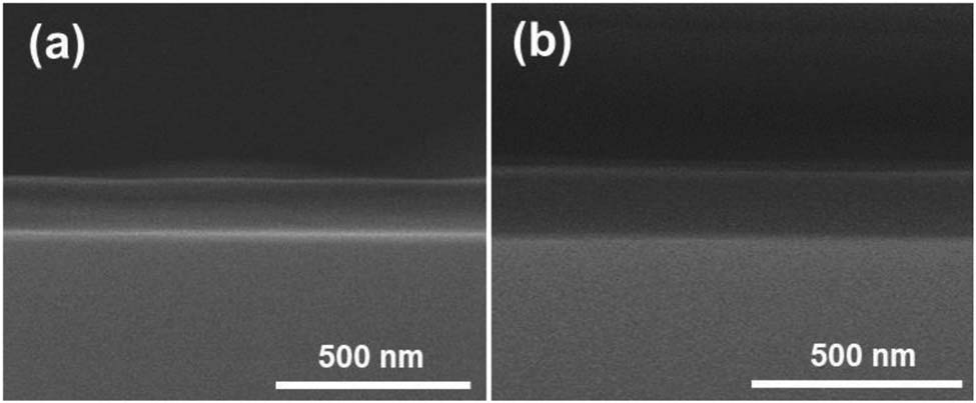
Cross-sectional SEM image of (a) PDDA/PVA(MMT) 30, (b) PDDA(GO)/PVA(MMT) 30 films.

Fig. 4 represents the thickness of the PDDA/PVA(MMT) 30 and PDDA(GO)/PVA(MMT) 30 multilayer. The thickness of the PDDA/PVA(MMT) 30 film is 126 nm and the thickness of PDDA(GO)/PVA(MMT) 30 film is 143 nm, indicating that there was no significant difference in thickness between the two films. This indicates that each layers PDDA(GO) and PVA(MMT) are well aligned. The AFM analysis also confirmed that the PDDA(GO)/PVA(MMT) film has the lowest roughness because each layer was well aligned.

**Fig. 5:**
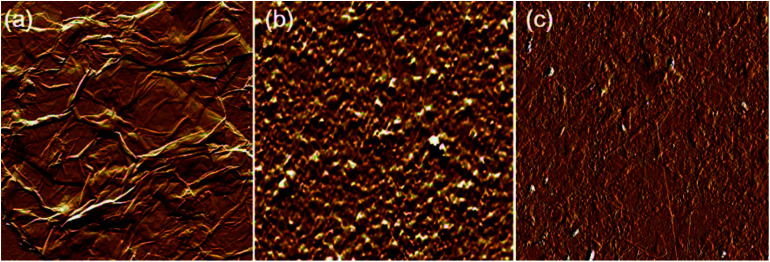
AFM images of (a) PDDA(GO), (b) PVA(MMT), and (c) PDDA(GO)/PVA(MMT) film.

The AFM analysis results of PDDA(GO), PVA(MMT), and PDDA(GO)/PVA(MMT) are given in Fig. 5. Surface roughnesses of PDDA(GO), PVA(MMT), and PDDA(GO)/PVA(MMT) films were approximately 0.929 nm, 0.128 nm, 0.095 nm. Because increases in the smoothness of the layer surface of the thin film correspond to improvements of gas-barrier properties, the PDDA(GO)/PVA(MMT) gas-barrier film may show the lowest WVTR values.^33,34^ These results confirm that each layer material can be stably bonded by electrostatic attractions. We compared the WVTR and transmittance values of 10 laminated layers of PDDA/PVA(MMT), and PDDA(GO)/PVA(MMT). The WVTR values of the gas barrier films were analyzed by the Ca-test method. In this system, the behavior of Ca changed from metallic to insulating when Ca reacted with H_2_O, with decreasing current under a constant applied voltage. The WVTR can be expressed as:
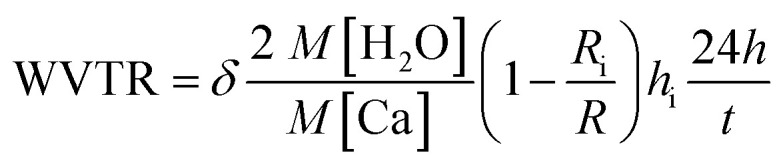
where *δ* is the Ca density, *M* is the molar mass of the indicated reagent, *R* is the resistance of the Ca connected to Ag electrodes, and *h* denotes the Ca height. *R*_i_ and *h*_i_ are the initial values of *R* and *h*, respectively. WVTR is proportional to the conductance, as indicated by a decrease in the Ca height Δ*h versus* the elapsed time Δ*t*.^35,36^

**Fig. 6:**
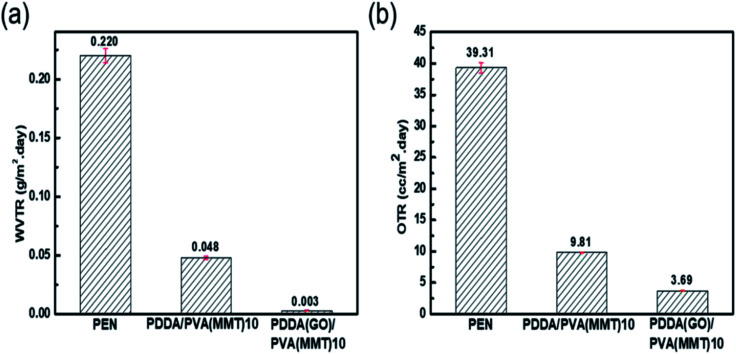
(a) WVTR and (b) OTR results of gas-barrier films.

Fig. 6(a) shows the WVTR of the uncoated PEN film of 2.2 × 10^−1^ g per m^2^ per day, that of the 10-layer PDDA(GO)-coated PEN film of 3.6 × 10^−2^ g per m^2^ per day, that of the 10-layer PDDA/PVA(MMT)-coated PEN film of 4.8 × 10^−2^ g per m^2^ per day, and that of the 10-layer PDDA(GO)/PVA(MMT)-coated PEN film being 3.1 × 10^−3^ g per m^2^ per day. When GO and MMT are alternately laminated, compared to when each is used alone, as shown in Scheme 2, extending the diffusion path length for gas molecules by inserting GO into the PDDA. Hence, WVTR value of the PDDA(GO)/PVA(MMT) films was greatly reduced. The OTR values of films were analyzed by MOCON. As shown in Fig. 6(b), the lowest OTR is measured for the film containing GO and MMT as alternating layers. The OTR of the PEN substrate is measured as 39.31 cc per m^2^ per day, which decreases to 6.91 cc per m^2^ per day, and 3.69 cc per m^2^ per day for the 10-layer PDDA/PVA(MMT)-, and 10-layer PDDA(GO)/PVA(MMT)-coated PEN substrates, respectively. This result shows that the oxygen permeability is decreased for the same reason causing the decreased WVTR, which is the lengthening of gas-molecule diffusion pathways.

**Fig. 7:**
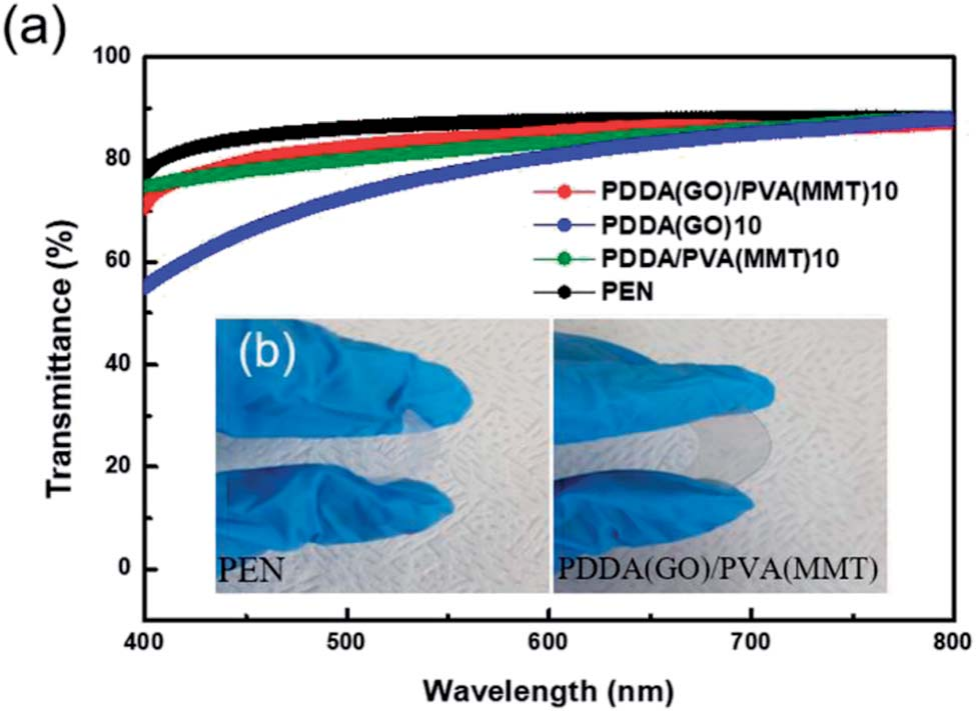
(a) Light transmittance of gas-barrier films fabricated by LBL process. (b) Optical images of PEN and 10-layer PDDA(GO)/PVA(MMT)-coated PEN films.

Since transmittance is an important factor in gas-barrier film, the light-transmittance properties of the gas-barrier films are measured using a UV-vis spectrophotometer. Fig. 7(a) shows at 550 nm, the transmittance values of uncoated PEN and 10-layer coatings of PDDA(GO), PDDA/PVA(MMT), and PDDA(GO)/PVA(MMT) films are 87.10% and 77.33%, 81.90%, and 84.05%, respectively. Since the GO–MMT film has the lowest roughness, the film comprising stacked GO and MMT shows the highest optical transmittance compared to the others.^34,37^ As shown in Fig. 7(b), the PDDA(GO)/PVA(MMT) flexible gas-barrier film is sufficiently transparent. This result shows that the characteristics of WVTR are further improved without significant deterioration in light transmittance by the alternating lamination of GO and MMT in films of the same number of layers.

**Fig. 8:**
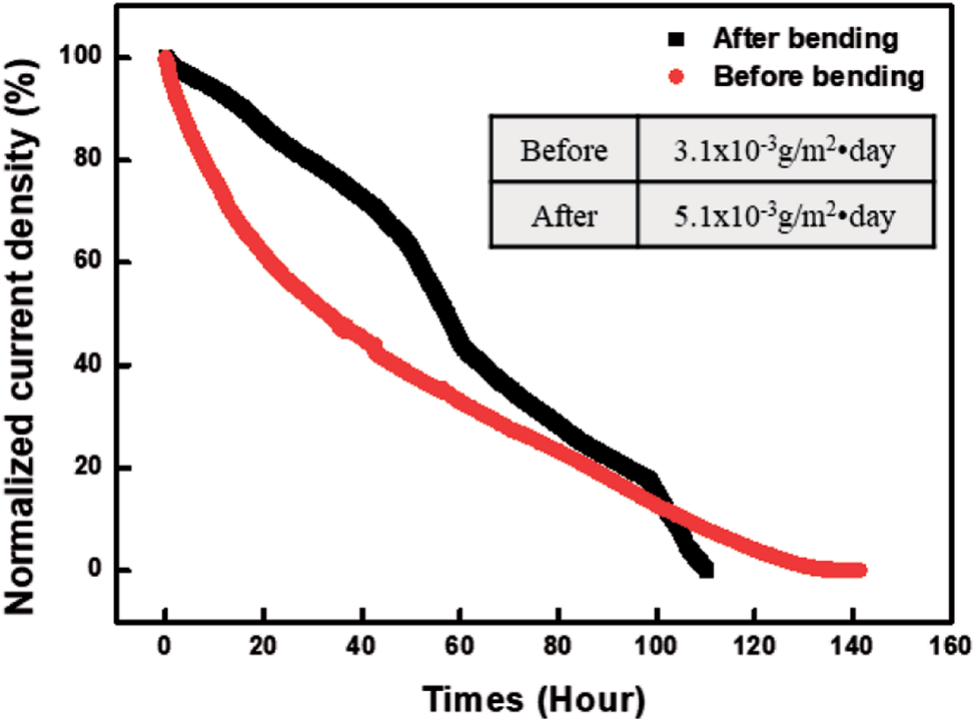
WVTR results before and after bending test of PDDA(GO)/PVA(MMT) assemblies on PEN.

Fig. 8 graphs a comparison of the WVTR values measured before and after the bending testing of the PDDA(GO)/PVA(MMT) gas-barrier film. The bending test was repeated 10 000 times to the minimum radius of 3 cm. The WVTR value is changed from 5.0 × 10^−3^ g per m^2^ per day before testing to 3.1 × 10^−3^ g per m^2^ per day afterward. This result shows that no significant change occurs in the WVTR value, even after repeated bending; therefore, this gas-barrier film is applicable to flexible electronic devices.^38^

## Conclusions

In this study, we used GO and MMT as alternative inorganic materials for a flexible gas-barrier thin film. The mixtures of PDDA(GO) and PVA(MMT) formed cationic and anionic complexes. Therefore, PDDA(GO) and PVA(MMT) were successfully deposited using the LBL method as a non-vacuum process to form a gas-barrier thin film with good interlayer bonding through electrostatic attraction. This method is cost-effective, short in fabrication time, and applicable to large-scale production. Our experimental results confirmed that a film comprising PDDA(GO)/PVA(MMT) multilayers showed decreased WVTR, better flexibility, and higher optical transmittance compared to single-material gas-barrier thin films without a large increase in thickness. The PDDA(GO)/PVA(MMT) multilayer-coated PEN gas-barrier thin film shows great potential for use in flexible applications.

## Conflicts of interest

There are no conflicts to declare.

## Supplementary Material

RA-008-C8RA08232D-s001
