# Correction: Fabrication of graphene oxide/montmorillonite nanocomposite flexible thin films with improved gas-barrier properties

**DOI:** 10.1039/c9ra90031d

**Published:** 2019-05-07

**Authors:** Se Jung Kim, Tan young Kim, Byung Hyun Kang, Gun-Hwan Lee, Byeong-Kwon Ju

**Affiliations:** Display and Nanosystem Laboratory, Department of Electrical Engineering, Korea University 145, Anam-ro, Seongbuk-gu Seoul 02841 Republic of Korea bkju@korea.ac.kr; Korea Institute of Materials Science, Surface Technology Division 797 Changwondaero Sungsangu Changwon Gyeongnam Korea ghlee@kims.re.kr

## Abstract

Correction for ‘Fabrication of graphene oxide/montmorillonite nanocomposite flexible thin films with improved gas-barrier properties’ by Se Jung Kim *et al.*, *RSC Adv.*, 2018, **8**, 39083–39089.

The authors regret that affiliation a was incorrect in the original article. The correct affiliations are as presented here.

The Royal Society of Chemistry apologises for these errors and any consequent inconvenience to authors and readers.

## Supplementary Material

