# Supplementary material for: Fabrication of graphene oxide/montmorillonite nanocomposite flexible thin films with improved gas-barrier properties
Source: RSC Adv. 2018 Nov 20;8(68):39083–9. doi: 10.1039/c8ra08232d (PMC9090656; doi:10.1039/c8ra08232d)
Supplement: RA-008-C8RA08232D-s001 [file RA-008-C8RA08232D-s001.pdf]

Electronic Supplementary Material (ESI) for RSC Advances.
This journal is © The Royal Society of Chemistry 2018

Supporting Information

Fabrication of Graphene Oxide/Montmorillonite nanocomposites flexible thin films with improved gas-barrier properties

*Se Jung Kim, Tan young Kim, Byung Hyun Kang, and Byeong-Kwon Ju**

1. WVTR measurement technique for the gas-barrier film

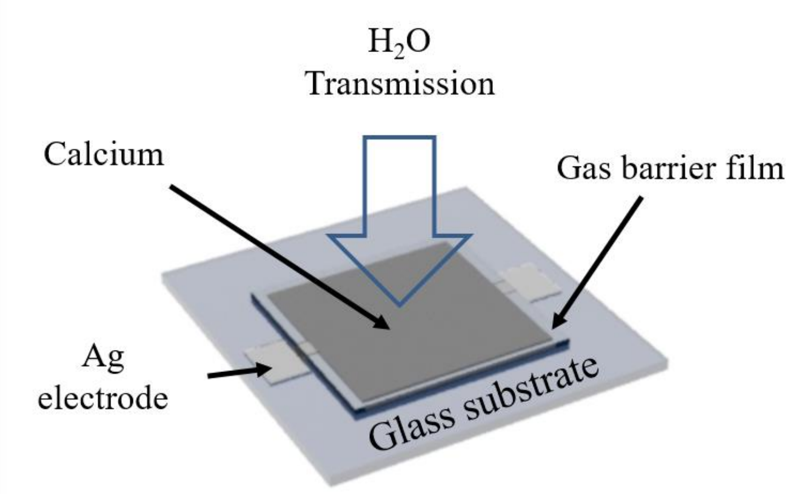

Figure. S1 Schematics for Ca-test system

WVTR values were analyzed using the Ca-test method, shown in Figure S1. A Ag electrode was deposited on a glass substrate and Ca was deposited on this to a height of 100 nm. The flexible gas-barrier film on the deposited Ca was attached using UV-resin. A Keithley 237 multimeter was used to measure the *I–V* characteristics. The voltage applied to the Ag electrode was 5mV. In accordance with previous studies, the WVTR value of the gas-barrier film was measured using the property that the resistance of Ca changes due to the role of the insulator when Ca reacts with H_2O.

2. Change in transmittance of a Ca test cell

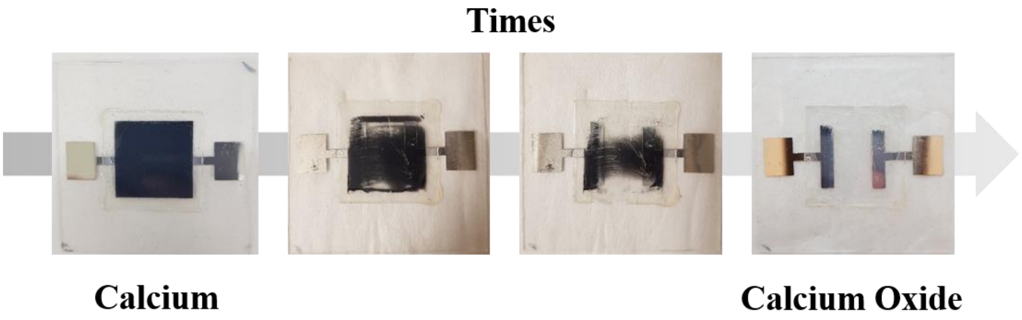

Figure. S2 Changes in Ca-test cell with time.

Ca is oxidized by water permeation through the gas-barrier film, according to the following chemical reactions:

$Ca + H_2O \rightarrow CaO + H_2$ (Equation S1)

$2Ca + O_2 \rightarrow 2CaO$ (Equation S2)

$CaO + H_2O \rightarrow Ca(OH)_2$ (Equation S3)

Figure. S2 shows that as Ca reacts with water permeating the gas-barrier film, the Ca is oxidized and its transmittance changes in accordance with time. Since CaO has a transparent characteristic, it was confirmed that Figure. S2 gradually became more transparent with time.
